# Characteristics and risk factors for infection and mortality caused by *Klebsiella pneumoniae* in patients with acute pancreatitis

**DOI:** 10.3389/fpubh.2024.1533765

**Published:** 2025-01-17

**Authors:** Jing Chen, Qian Xiang, Jia-yu Wu, Xiao-Jia Zheng, Xiao-yan Jiang

**Affiliations:** Department of Healthcare-Associated Infection Control Center, Sichuan Provincial People's Hospital, University of Electronic Science and Technology of China, Chengdu, China

**Keywords:** acute pancreatitis, *Klebsiella pneumoniae*, carbapenem resistance, risk factor, mortality

## Abstract

**Background:**

The purpose of this study is to investigate the risk factors for carbapenem-resistant *Klebsiella pneumoniae* (CRKP) infection and death of *K. pneumoniae* infections in acute pancreatitis (AP) patients.

**Methods:**

This was a retrospective analysis of AP patients with *K. pneumoniae* infection between January 2019 and April 2024. Logistic regression model was used to determine the risk factors for acquisition of CRKP. Cox proportional hazards regression model was used to identify risk factors for mortality of *K. pneumoniae* infection.

**Results:**

A total of 113 AP patients experienced *K. pneumoniae* infections, including 66 with CRKP infection and 47 with CSKP infection. The mortality rate of these patients was 36.3%, with a significantly higher mortality rate in the CRKP group than in the CSKP group (*P* < 0.001). Only mechanical ventilation (OR = 2.301; 95% CI 1.02-5.20; *P* = 0.045) was the independent risk factor for CRKP infection in patients with AP. Surgery (HR 0.38; 95% CI 0.20–0.75; *P* = 0.005) and mechanical ventilation (HR 2.93; 95% CI 1.10–7.82; *P* = 0.032) were the independent risk factors associated with mortality of *K. pneumoniae* infection in patients with AP.

**Conclusion:**

*Klebsiella pneumoniae* infection has become a threat to patients with AP. Therefore, implementing preventive and control measures based on risk factors is crucial for the prognosis.

## Introduction

Acute pancreatitis (AP) is a common acute abdominal disease, and the incidence rate continues to increase worldwide (1). Severe acute pancreatitis (SAP) is a severe form of AP with systemic organ failure and a mortality rate of up to 30% (2). Infection is a common complication of AP, with nearly a third of patients with AP developing pancreatic or extra-pancreatic infection (3). Secondary infection may be an independent risk factor for poor prognosis in AP (4).

Currently, carbapenem-resistant *Klebsiella pneumoniae* (CRKP) has become the main nosocomial pathogen, which can cause various types of infections, such as pneumonia, bloodstream infections (BSI), intra-abdominal infections, and urinary tract infections (5). In recent years, the rate of isolation and reporting of CRKP infections has been on the rise globally, with a particularly prominent increase in Asia compared to Europe and the United States (6). CRKP infections have become a significant public health threat, with limited options for antimicrobial treatment, posing a great challenge to both treatment and prevention and control. *Klebsiella pneumoniae* is a common pathogen of infection in patients with AP. The treatment of AP complicated by CRKP infection is more difficult, resulting in higher mortality rates, longer hospital stays, and a severely compromised prognosis for the patients (7). Previous studies have shown that complications caused by multidrug-resistant bacterial infections increase the mortality rate of AP patients (6, 8, 9). Although strict prevention and control measures have been taken in hospitals for patients with AP, the occurrence of CRKP infection still exists. Therefore, patients with AP complicated by CRKP infection deserve attention. This retrospective cohort study aims to investigate the risk factors for AP patients complicated with CRKP infection, in order to help clinicians better take prevention and control measures, and improve clinical outcomes for these patients in the future.

## Methods

### Study design and participants

This retrospective cohort study was conducted in Sichuan Academy of Medical Sciences and Sichuan Provincial People's Hospital, a 4,300-bed teaching hospital in Western China. The clinical data of AP patients with *K. pneumoniae* infection admitted from January 2019 to April 2024 was collected. Patients with *K. pneumoniae* isolated from any clinical specimen within 48 h after admission and those under the age of 18 were excluded. Patients with incomplete clinical data were also excluded. Only the first episode of *K. pneumoniae* infection was included during the study period. This study was approved by the Ethics Committee of Sichuan Academy of Medical Sciences and Sichuan Provincial People's Hospital. The ethics committee exempted the requirement for informed consent as it was a retrospective study.

### Microbiology

The sensitivity of carbapenem was determined by agar dilution method and interpreted referring to guidelines of the Clinical and Laboratory Standards Institute (CLSI) (10). Disk-diffusion synergistic test recommended by CLSI was used for screening and detecting *K. pneumoniae*. The automated instrument used the VITEK 2 (BioMerieux, France) compact automatic microbial identification and drug sensitivity analysis system. The disk-diffusion synergistic test adopted the Kirby Bauer method, and the drug sensitive paper was sourced from BBL and Oxoid. Consistent with the CLSI definition, CRKP was defined as *K. pneumoniae* with a minimum inhibitory concentration (MIC) of ≥2 mg/L for ertapenem or a MIC of ≥4 mg/L for meropenem or imipenem or dolipenem (10).

### Data collection

Baseline characteristics of patients were collected from the electronic medical record of the first episode of *K. pneumoniae*, including age, gender, body mass index (BMI), comorbidities, intravenous catheter, mechanical ventilation, urinary catheterization, surgery, ICU exposure, length of hospitalization, type of infection and mortality, etc. Microbiological and laboratory data were collected from the laboratory information system (LIS), including white blood cells (WBC), hemoglobin, platelet count, C-reactive protein, serum creatinine, albumin, total bilirubin, blood glucose, and procalcitonin (PCT). The data would be entered into an Excel spreadsheet and verified by two dedicated infection control personnel after collection. According to the death events during hospitalization, clinical outcomes were divided into survival group and non-survival group.

### Statistical analysis

Quantitative data are represented as mean ± standard deviation or median (range), depending on whether it conforms to normal distribution. The student's *t*-test or Mann-Whitney U test (as appropriate) was performed to analyze the differences between groups. Categorical variables were expressed in absolute numbers and percentages, and compared with the χ^2^ test or Fisher exact test. AP patients with *K. pneumoniae* infection were divided into two groups based on whether they were resistant to carbapenem antibiotics: CRKP infection group and CSKP infection group. We used univariate and multivariate binary logistic regression to identify risk factors for CRKP infection. Univariate and multivariate Cox proportional hazards regression models are used to study independent risk factors for mortality. The hazard ratio (HR) and 95% confidence interval (CI), as well as the corresponding *P*-value are provided. To evaluate the validity of the proportional hazards assumption, Schoenfeld residual tests were further conducted. For continuous variables such as PCT and WBC, appropriate cut-off values were determined based on clinical relevance and previous literature (10, 11). No data transformations were performed in our analysis. All statistical analyses were performed using SPSS 27.0 and R 4.4.2 statistical software. *P* < 0.05 was considered statistically significant.

## Results

### Baseline characteristics of patients with AP complicated by *K. pneumoniae* infection

In the past 5 years, a total of 113 AP patients experienced *K. pneumoniae* infections, including 66 with CRKP infection and 47 with CSKP infection. The average age was 57 years old, with a predominance of males (65.5%). Most isolates were isolated from the respiratory system (35.4%), followed by bloodstream (23.0%) and abdominal cavity (19.5%). There were 60 patients (53.1%) with SAP. Among the 126 patients, 74.3% were admitted to the intensive care unit (ICU) due to worsening conditions. The mortality rate in the CRKP group was significantly higher than that in the CSKP group (*P* < 0.001). The demographic and clinical characteristics of AP patients with *K. pneumoniae* infection are shown in Table 1.

**Table 1:** The demographic and clinical characteristics of AP patients with *K. pneumoniae* infection.

**Characteristic**	**Total (n = 113)**	**CSKP infection group (n = 47)**	**CRKP infection group (n = 66)**	**P value**
Gender, male, *n* (%)	74 (65.5)	32 (68.1)	42 (63.6)	0.624
Age, mean ± SD	57.0 ± 18.1	59.5 ± 17.4	55.2 ± 18.6	0.215
BMI, mean ± SD	23.8 ± 4.2	23.4 ± 4.2	24.0 ± 4.2	0.470
**Underlying diseases**, ***n*** **(%)**				
Hypertension	8 (7.1)	5 (10.6)	3 (4.5)	0.274
Diabetes	18 (15.9)	9 (19.1)	9 (13.6)	0.430
SAP, *n* (%)	60 (53.1)	18 (38.3)	42 (63.6)	0.008
Surgery, *n* (%)	50 (44.2)	22 (46.8)	28 (42.4)	0.644
Admission to ICU, *n* (%)	84 (74.3)	31 (66)	53 (80.3)	0.085
Blood transfusion, *n* (%)	78 (69.0)	32 (68.1)	46 (69.7)	0.855
Urinary catheter, *n* (%)	58 (51.3)	23 (48.9)	35 (53)	0.668
Intravascular catheter, *n* (%)	74 (65.5)	28 (59.6)	46 (69.7)	0.265
Mechanical ventilation, *n* (%)	58 (51.3)	17 (36.2)	41 (62.1)	0.007
**Main types of infections**, ***n*** **(%)**				
Respiratory infection	40 (35.4)	16 (34)	24 (36.4)	0.799
Bloodstream infection	26 (23.0)	15 (31.9)	11 (16.7)	0.058
Intra-abdominal infection	22 (19.5)	9 (19.1)	13 (19.7)	0.942
Mortality, *n* (%)	41 (36.3)	8 (17)	33 (50)	< 0.001

AP, acute pancreatitis; CSKP, carbapenem- sensitive *Klebsiella pneumoniae*; CRKP, carbapenem-resistant *Klebsiella pneumoniae*; SD, standard deviation; BMI, body mass index; SAP, severe acute pancreatitis; ICU, intensive care unit.

### Risk factors associated with CRKP infection in patients with AP

Both the CRKP infection group and CSKP infection group were predominantly male patients, and their ages were comparable. The proportion of SAP and mechanical ventilation in the CRKP infection group was significantly higher than that in the CSKP infection group (*P* < 0.05). The proportion of patients admitted to ICU in the CRKP infection group was also higher, but the difference had no statistical significance. The univariate logistic analysis of demographic and clinical characteristics of patients with *K. pneumoniae* infection is shown in Table 1. After logistic multivariate analysis, only mechanical ventilation (OR = 2.301; 95% CI 1.02–5.20; *P* = 0.045) was the independent risk factor for CRKP infection in patients with AP (Table 2).

**Table 2:** Multivariate analysis of risk factors associated with CRKP infection in patients with AP.

**Variables**	***P* value**	**OR**	**95%CI**
SAP	0.055	2.219	(0.98, 5.01)
Mechanical ventilation	0.045	2.301	(1.02, 5.20)

CRKP, carbapenem-resistant *Klebsiella pneumoniae*; AP, acute pancreatitis; SAP, severe acute pancreatitis; OR, odds ratio; CI, confidence interval.

### Risk factors for mortality of *K. pneumoniae* infection in patients with AP

Among patients with AP complicated by *K. pneumoniae* infection, 41 patients died, with a mortality rate of 36.3%. Cox univariate analysis showed that there was a statistically significant difference in age >60 years old, carbapenem resistance, surgery, mechanical ventilation, and PCT >5 ng/ml between the death and survival groups. In Cox multivariate analysis, surgery (HR 0.38; 95% CI 0.20–0.75; *P* = 0.005) and mechanical ventilation (HR 2.93; 95% CI 1.10–7.82; *P* = 0.032) were the independent risk factors associated with mortality (Table 3). To evaluate the effectiveness of the Cox proportional hazards model, Schoenfeld residual test was used for validation. The results showed no significant violation of the proportional hazards assumption (*P* = 0.392) (Supplementary Table S1, Supplementary Figure S1).

**Table 3:** Univariate and multivariate analysis of the risk factors for mortality of KP infection in patients with AP.

**Variable**	**Univariate analysis**		**Multivariable analysis**	
	**HR (95%CI)**	***P*** **value**	**HR (95%CI)**	***P*** **value**
Male	1.99 (0.97, 4.08)	0.061		
Age > 60 years old	2.26 (1.21, 4.25)	0.011	1.84 (0.94~3.63)	0.075
BMI > 24 kg/m^2^	0.62 (0.33, 1.15)	0.128		
Carbapenem resistance	2.63 (1.21, 5.70)	0.015	1.92 (0.86~4.26)	0.111
**Underlying diseases**				
Hypertension	1.70 (0.4, 7.26)	0.472		
Diabetes	0.18 (0.02, 1.34)	0.095		
SAP	0.83 (0.45, 1.56)	0.570		
Surgery	0.31 (0.16, 0.60)	< 0.001	0.38 (0.20~0.75)	0.005
Admission to ICU	1.29 (0.49, 3.38)	0.607		
Blood transfusion	1.20 (0.48, 2.99)	0.689		
Urinary catheter	0.58 (0.30, 1.12)	0.103		
Intravascular catheter	1.09 (0.46, 2.58)	0.847		
Mechanical ventilation	3.69 (1.53, 8.89)	0.004	2.93 (1.10~7.82)	0.032
**Laboratory test**				
PCT > 5 ng/ml	2.23 (1.03, 4.85)	0.043	0.97 (0.39~2.41)	0.947
HB < 90 g/L	0.98 (0.47, 2.04)	0.955		
Creatinine > 177 umol/L	1.78 (0.94, 3.35)	0.075		
Albumin < 30 g/L	1.43 (0.55, 3.72)	0.461		
Glucose > 10 mmol/L	0.68 (0.27, 1.77)	0.434		
Heart failure	1.06 (0.44, 2.57)	0.895		
Liver failure	0.79 (0.28, 2.24)	0.663		
Renal failure	1.38 (0.58, 3.29)	0.466		
ICU stay > 7 days	1.48 (0.77, 2.81)	0.237		

KP, *Klebsiella pneumoniae*; AP, acute pancreatitis; HR, adjusted hazard ratio; CI, confidence interval; BMI, body mass index; SAP, severe acute pancreatitis; ICU, intensive care unit; PCT, procalcitonin; HB, hemoglobin.

### Risk factors for mortality of CRKP infection in patients with AP

Among 66 patients infected with CRKP, 33 died with a mortality rate of 50%. In the Cox univariate analysis, male, age > 60 years old, surgery, mechanical ventilation and renal failure were associated with the mortality of CRKP infection. After Cox multivariate analysis, male (HR 2.73; 95% CI 1.23–6.08; *P* = 0.014), age > 60 years old (HR 2.00; 95% CI 1.03–3.88; *P* = 0.041), surgery (HR 0.32; 95% CI 0.16–0.64; *P* = 0.001) and mechanical ventilation (HR 2.82; 95% CI 1.05–7.55; *P* = 0.040) were independent risk factors for mortality of CRKP infection (Table 4). The Schoenfeld residuals test showed no significant violations of the proportional hazards assumption (*P* = 0.190) (Supplementary Table S2, Supplementary Figure S2).

**Table 4:** Risk factors for mortality of CRKP infection in AP patients with univariate and multivariate analysis.

**Variable**	**Univariate analysis**		**Multivariable analysis**	
	**HR (95%CI)**	***P*** **value**	**HR (95%CI)**	***P*** **value**
Male	3.21 (1.32, 7.84)	0.010	2.73 (1.23~6.08)	0.014
Age > 60 years old	2.74 (1.34, 5.61)	0.006	2.00 (1.03~3.88)	0.041
BMI > 24 kg/m^2^	0.52 (0.26, 1.05)	0.068		
Hypertension	2.68 (0.33, 21.44)	0.354		
SAP	0.58 (0.29, 1.17)	0.131		
Surgery	0.37 (0.18, 0.77)	0.007	0.32 (0.16~0.64)	0.001
Admission to ICU	1.36 (0.40, 4.58)	0.623		
Blood transfusion	0.85 (0.33, 2.16)	0.733		
Urinary catheter	0.60 (0.29, 1.23)	0.159		
Intravascular catheter	0.97 (0.35, 2.69)	0.951		
Mechanical ventilation	3.18 (1.10, 9.18)	0.033	2.82 (1.05~7.55)	0.040
**Laboratory test**				
PCT > 5 ng/ml	1.79 (0.74, 4.37)	0.199		
HB < 90 g/L	0.67 (0.30, 1.49)	0.321		
Creatinine > 177 umol/L	1.51 (0.73, 3.16)	0.268		
Albumin < 30 g/L	0.93 (0.32, 2.76)	0.901		
Glucose > 10 mmol/L	0.72 (0.27, 1.91)	0.507		
Heart failure	1.32 (0.50, 3.49)	0.580		
Liver failure	1.24 (0.37, 4.13)	0.725		
Renal failure	3.06 (1.04, 9.02)	0.042	1.41 (0.64~3.10)	0.400
ICU stay > 7 days	1.06 (0.50, 2.23)	0.881		

CRKP, carbapenem-resistant *Klebsiella pneumoniae*; AP, acute pancreatitis; HR, adjusted hazard ratio; CI, confidence interval; BMI, body mass index; SAP, severe acute pancreatitis; ICU, intensive care unit; PCT, procalcitonin; HB, hemoglobin.

## Discussion

The rapid emergence of multidrug-resistant bacteria in the past decade has become a global threat to public health system (12). In recent years, with the overuse of antibiotics, the incidence of CRKP has increased and has become an important pathogen of hospital-acquired infections. CRKP has been listed as one of the key priority bacteria by the World Health Organization, which would increase mortality and make treatment more difficult (13, 14). Infection is one of the most common complications in AP patients (15). Due to the early and widespread use of antibiotics, patients with AP have become a high-risk group for CRKP infection. Previous study found that carbapenem-resistant *Enterobacteriaceae* infection was a key factor in the mortality of AP patients in ICU (16). In this study, the mortality rate among patients with AP complicated by *K. pneumoniae* infection was 36.3%, while the mortality rate reached 50% in those complicated by CRKP infection, which was similar to previous reports (10, 17).

The results showed that the most common site of infection was lower respiratory tract infection, with 51.3% of *K. pneumoniae* infected patients received mechanical ventilation. Moreover, multivariate analysis showed that mechanical ventilation was the independent risk factor for CRKP infection, indicating that drug-resistant bacteria was prone to invade the respiratory tract. Therefore, infection prevention and control measures should be taken to prevent cross infection during tracheal intubation.

This study suggested that surgery and mechanical ventilation were independent risk factors for death in *K. pneumoniae* infection. Carbapenems were once considered the most effective drugs against multidrug-resistant Gram-negative pathogens (18, 19). However, the widespread use of carbapenem has led to an increase in resistant gram-negative bacteria. The emergence of drug-resistant bacteria significantly limited the available therapeutic options, and affected the prognosis. Our results showed that the mortality rate of CRKP infected patients was 50%, significantly higher than that of CSKP infected patients, which was consistent with previous research finding (20). Therefore, strict infection control measures and reasonable antibiotic selection should be taken to reduce the occurrence of CRKP infection. In this study, surgery was a protective factor for the prognosis of AP patients with KP infection. For patients with SAP combined with peripheral tissue necrosis and infection, surgery is the main treatment method at present. Surgical removal of necrotic tissue can clear the infection site, which is beneficial for infection control and patient recovery. However, there is still controversy over whether AP patients need surgery and the surgical methods used. The current consensus is that surgery should be performed on patients with clinically unstable infectious necrosis (21). For most AP patients with sterile inflammation, conservative treatment can be administered regardless of the amount and extent of exudate (22). The decision to perform surgery in AP patients should be carefully weighed against the potential benefits and risks. For patients who need surgery, the surgical risk should be assessed before surgery, appropriate surgical methods should be adopted, and infection monitoring should be done after surgery to reduce the infection rate. Mechanical ventilation, while providing therapeutic benefits to patients, may also affect the prognosis of critically ill patients by affecting the function of various organs (23). Therefore, careful management of ventilation is crucial. For patients undergoing mechanical ventilation, it is advisable to minimize the duration of mechanical ventilation and implement standardized infection control measures such as oral care and elevated bed heads to reduce the risk of respiratory infections and improve prognosis.

There were several potential limitations in our study. Firstly, this was a single-center retrospective cohort study with a relatively small sample size, so the results may differ from other studies. Further multicenter and prospective studies are needed. Secondly, this study did not involve antibiotic treatment or other factors, and further large-scale studies are needed to include more factors to determine the risk factors for *K. pneumoniae* occurrence or death in AP patients.

## Conclusions

In conclusion, *K. pneumoniae* is a common pathogen of infection in patients with AP. In our research, only mechanical ventilation was the independent risk factor for CRKP infection in AP patients. Surgery and mechanical ventilation were independent risk factors for death in AP patients with *K. pneumoniae* infection. It is necessary to take corresponding prevention and control measures based on these risk factors to reduce the occurrence of drug-resistant bacterial infection.

## Data Availability

The original contributions presented in the study are included in the article/Supplementary material, further inquiries can be directed to the corresponding author.
