# Supplementary material for: Characteristics and risk factors for infection and mortality caused by Klebsiella pneumoniae in patients with acute pancreatitis
Source: Front Public Health. 2025 Jan 17;12:1533765. doi: 10.3389/fpubh.2024.1533765 (PMC11782239; doi:10.3389/fpubh.2024.1533765)
Supplement: Supplementary file 1 [file Data_Sheet_1.docx]

Tables

Table S1 Schoenfeld residuals test for proportional hazards assumption in cox regression model for mortality risk factors of K. pneumoniae infection in AP patients

| Variable | Test statistic | P value |
| --- | --- | --- |
| Age>60 years old | 1.001 | 0.317 |
| Carbapenem resistance | 0.962 | 0.327 |
| Surgery | 3.040 | 0.081 |
| Mechanical ventilation | 0.175 | 0.675 |
| PCT>5 ng/ml | 0.186 | 0.666 |
| Global | 5.196 | 0.392 |

Abbreviations: AP, acute pancreatitis; PCT, procalcitonin.

Table S2 Schoenfeld residuals test for proportional hazards assumption in cox regression model for mortality risk factors of CRKP infection in AP patients

| Variable | Test statistic | P value |
| --- | --- | --- |
| Male | 0.156 | 0.69 |
| Age>60 years old | 0.301 | 0.58 |
| Surgery | 1.151 | 0.28 |
| Mechanical ventilation | 0.013 | 0.91 |
| Renal failure | 0.876 | 0.35 |
| Global | 7.444 | 0.19 |

Abbreviations: CRKP, carbapenem-resistant *Klebsiella pneumoniae*; AP, acute pancreatitis.
