# Supplementary material for: Characteristics and risk factors for infection and mortality caused by Klebsiella pneumoniae in patients with acute pancreatitis
Source: Front Public Health. 2025 Jan 17;12:1533765. doi: 10.3389/fpubh.2024.1533765 (PMC11782239; doi:10.3389/fpubh.2024.1533765)
Supplement: Supplementary file 2 [file Data_Sheet_2.docx]

**Code for Statistical Analysis**

**Table 1:**

* This section generated the descriptive statistics (SPSS) for Table 1.

GET DATA

  /TYPE=XLS

  /FILE='D:\胰腺炎合并肺克\分析\投稿\AP with KP.xls'

  /SHEET=name 'Sheet1'

  /CELLRANGE=full

  /READNAMES=on

   /DATATYPEMIN PERCENTAGE=95.0.

EXECUTE.

DATASET NAME DataSet1 WINDOW=FRONT.

CROSSTABS

  /TABLES=group BY gender hypertension diabetes SAP surgery admission to ICU blood transfusion urinary catheter CVC mechanical ventilation respiratory infection bloodstream infection intra-abdominal infection mortality

  /FORMAT=AVALUE TABLES

  /STATISTICS=CHISQ

  /CELLS=COUNT

  /COUNT ROUND CELL.

T-TEST GROUPS=group(0 1)

/MISSING=ANALYSIS

/VARIABLES=age BMI

/ES DISPLAY(TRUE)

/CRITERIA=CI(.95).

**Table 2:**

* This section conducted multivariate logistic regression analysis (SPSS).

LOGISTIC REGRESSION VARIABLES group

/METHOD=ENTER SAP mechanical ventilation

/PRINT=GOODFIT SUMMARY CI(95)

/CRITERIA=PIN(0.05) POUT(0.10) ITERATE(20) CUT(0.5).

**Table 3:**

* This section used univariate and multivariate Cox regression analysis (R software).

install.packages("readxl")

install.packages("survival")

library(readxl)

library(survival)

file_path <- "D:/胰腺炎合并肺克/分析/投稿/AP with KP.xls"

data <- read_excel(file_path, sheet = 1)

head(data)

cox_model_gender <- coxph(Surv(time, status) ~ gender, data = data)

summary(cox_model_gender)

cox_model_age大于60 <- coxph(Surv(time, status) ~ age大于60, data = data)

summary(cox_model_age大于60)

cox_model_BMI大于24 <- coxph(Surv(time, status) ~ BMI大于24, data = data)

summary(cox_model_BMI大于24)

cox_model_carbapenem resistance <- coxph(Surv(time, status) ~ carbapenem resistance, data = data)

summary(cox_model_carbapenem resistance)

cox_model_hypertension <- coxph(Surv(time, status) ~ hypertension, data = data)

summary(cox_model_hypertension)

cox_model_diabetes <- coxph(Surv(time, status) ~ diabetes, data = data)

summary(cox_model_diabetes)

cox_model_SAP <- coxph(Surv(time, status) ~ SAP, data = data)

summary(cox_model_SAP)

cox_model_surgery <- coxph(Surv(time, status) ~ surgery, data = data)

summary(cox_model_surgery)

cox_model_admission to ICU <- coxph(Surv(time, status) ~ admission to ICU, data = data)

summary(cox_model_admission to ICU)

cox_model_blood transfusion <- coxph(Surv(time, status) ~ blood transfusion, data = data)

summary(cox_model_blood transfusion)

cox_model_urinary catheter <- coxph(Surv(time, status) ~ urinary catheter, data = data)

summary(cox_model_urinary catheter)

cox_model_CVC <- coxph(Surv(time, status) ~ CVC, data = data)

summary(cox_model_CVC)

cox_model_mechanical ventilation <- coxph(Surv(time, status) ~ mechanical ventilation, data = data)

summary(cox_model_mechanical ventilation)

cox_model_PCT大于5 <- coxph(Surv(time, status) ~ PCT大于5, data = data)

summary(cox_model_PCT大于5)

cox_model_HB小于90 <- coxph(Surv(time, status) ~ HB小于90, data = data)

summary(cox_model_HB小于90)

cox_model_crea大于177 <- coxph(Surv(time, status) ~ crea大于177, data = data)

summary(cox_model_crea大于177)

cox_model_ALB小于30 <- coxph(Surv(time, status) ~ ALB小于30, data = data)

summary(cox_model_ALB小于30)

cox_model_glu大于10 <- coxph(Surv(time, status) ~ glu大于10r, data = data)

summary(cox_model_glu大于10)

cox_model_heart failure <- coxph(Surv(time, status) ~ heart failure, data = data)

summary(cox_model_heart failure)

cox_model_liver failure <- coxph(Surv(time, status) ~ liver failure, data = data)

summary(cox_model_liver failure)

cox_model_renal failure <- coxph(Surv(time, status) ~ renal failure, data = data)

summary(cox_model_renal failure)

cox_model_ICU大于7d <- coxph(Surv(time, status) ~ ICU大于7d, data = data)

summary(cox_model_ICU大于7d)

cox_model <- coxph(Surv(time, status) ~ age大于60 + carbapenem resistance + surgery + mechanical ventilation + PCT大于5, data = data)

summary(cox_model)

schoenfeld_test <- cox.zph(cox_model)

print(schoenfeld_test)

plot(schoenfeld_test)

**Table 4:**

* This section used univariate and multivariate Cox regression analysis (R software).

file_path <- "D:/胰腺炎合并肺克/分析/投稿/CRKP.xls"

cox_model_gender <- coxph(Surv(time, status) ~ gender, data = data)

summary(cox_model_gender)

cox_model_age大于60 <- coxph(Surv(time, status) ~ age大于60, data = data)

summary(cox_model_age大于60)

cox_model_BMI大于24 <- coxph(Surv(time, status) ~ BMI大于24, data = data)

summary(cox_model_BMI大于24)

cox_model_carbapenem resistance <- coxph(Surv(time, status) ~ carbapenem resistance, data = data)

summary(cox_model_carbapenem resistance)

cox_model_hypertension <- coxph(Surv(time, status) ~ hypertension, data = data)

summary(cox_model_hypertension)

cox_model_diabetes <- coxph(Surv(time, status) ~ diabetes, data = data)

summary(cox_model_diabetes)

cox_model_SAP <- coxph(Surv(time, status) ~ SAP, data = data)

summary(cox_model_SAP)

cox_model_surgery <- coxph(Surv(time, status) ~ surgery, data = data)

summary(cox_model_surgery)

cox_model_admission to ICU <- coxph(Surv(time, status) ~ admission to ICU, data = data)

summary(cox_model_admission to ICU)

cox_model_blood transfusion <- coxph(Surv(time, status) ~ blood transfusion, data = data)

summary(cox_model_blood transfusion)

cox_model_urinary catheter <- coxph(Surv(time, status) ~ urinary catheter, data = data)

summary(cox_model_urinary catheter)

cox_model_CVC <- coxph(Surv(time, status) ~ CVC, data = data)

summary(cox_model_CVC)

cox_model_mechanical ventilation <- coxph(Surv(time, status) ~ mechanical ventilation, data = data)

summary(cox_model_mechanical ventilation)

cox_model_PCT大于5 <- coxph(Surv(time, status) ~ PCT大于5, data = data)

summary(cox_model_PCT大于5)

cox_model_HB小于90 <- coxph(Surv(time, status) ~ HB小于90, data = data)

summary(cox_model_HB小于90)

cox_model_crea大于177 <- coxph(Surv(time, status) ~ crea大于177, data = data)

summary(cox_model_crea大于177)

cox_model_ALB小于30 <- coxph(Surv(time, status) ~ ALB小于30, data = data)

summary(cox_model_ALB小于30)

cox_model_glu大于10 <- coxph(Surv(time, status) ~ glu大于10r, data = data)

summary(cox_model_glu大于10)

cox_model_heart failure <- coxph(Surv(time, status) ~ heart failure, data = data)

summary(cox_model_heart failure)

cox_model_liver failure <- coxph(Surv(time, status) ~ liver failure, data = data)

summary(cox_model_liver failure)

cox_model_renal failure <- coxph(Surv(time, status) ~ renal failure, data = data)

summary(cox_model_renal failure)

cox_model_ICU大于7d <- coxph(Surv(time, status) ~ ICU大于7d, data = data)

summary(cox_model_ICU大于7d)

cox_model <- coxph(Surv(time, status) ~ gender + age大于60 + surgery + mechanical ventilation + renal failure, data = data)

summary(cox_model)

schoenfeld_test <- cox.zph(cox_model)

print(schoenfeld_test)

plot(schoenfeld_test)
