# Supplementary figures and images for: Characteristics and risk factors for infection and mortality caused by Klebsiella pneumoniae in patients with acute pancreatitis
Source: Front Public Health. 2025 Jan 17;12:1533765. doi: 10.3389/fpubh.2024.1533765 (PMC11782239; doi:10.3389/fpubh.2024.1533765)

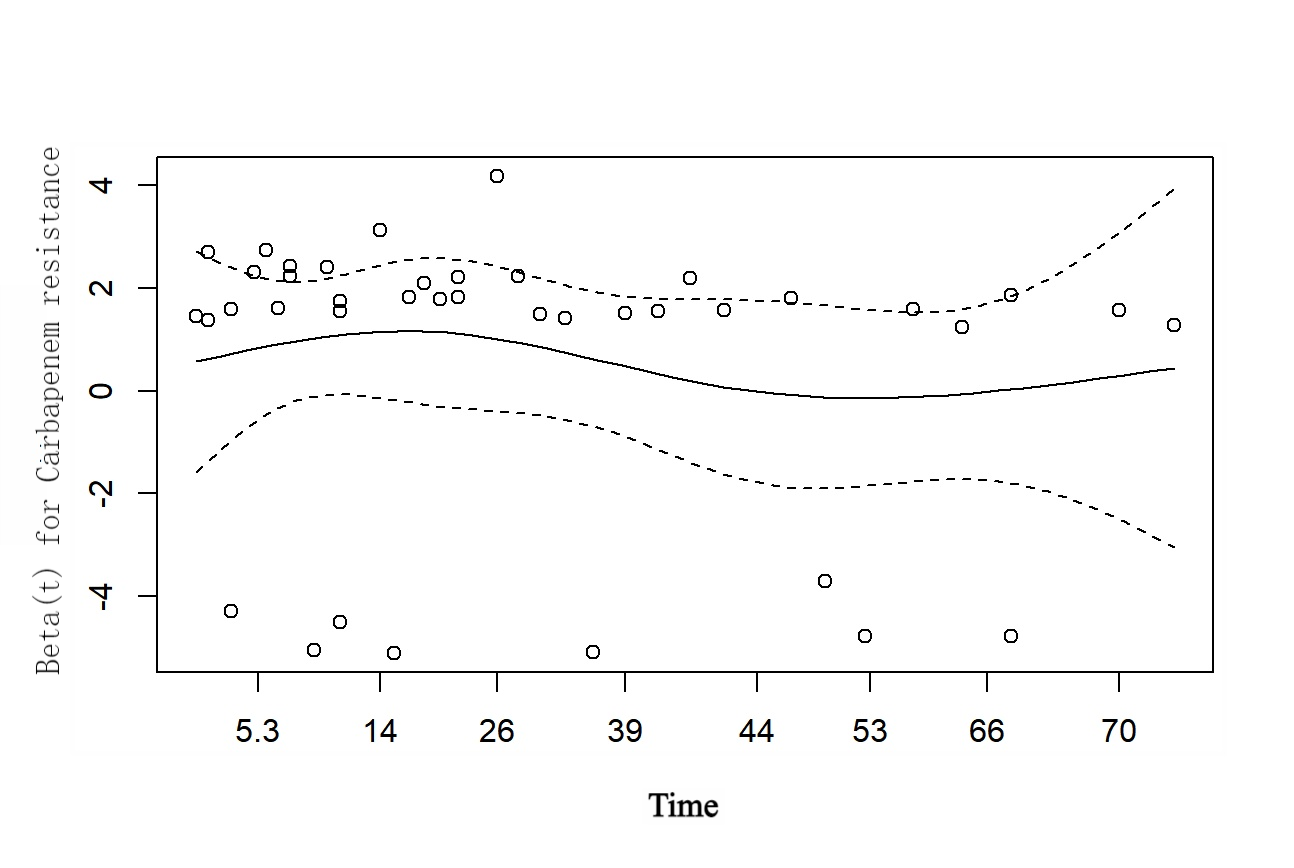

Supplement: Supplementary file 3 [file Image_1.png]

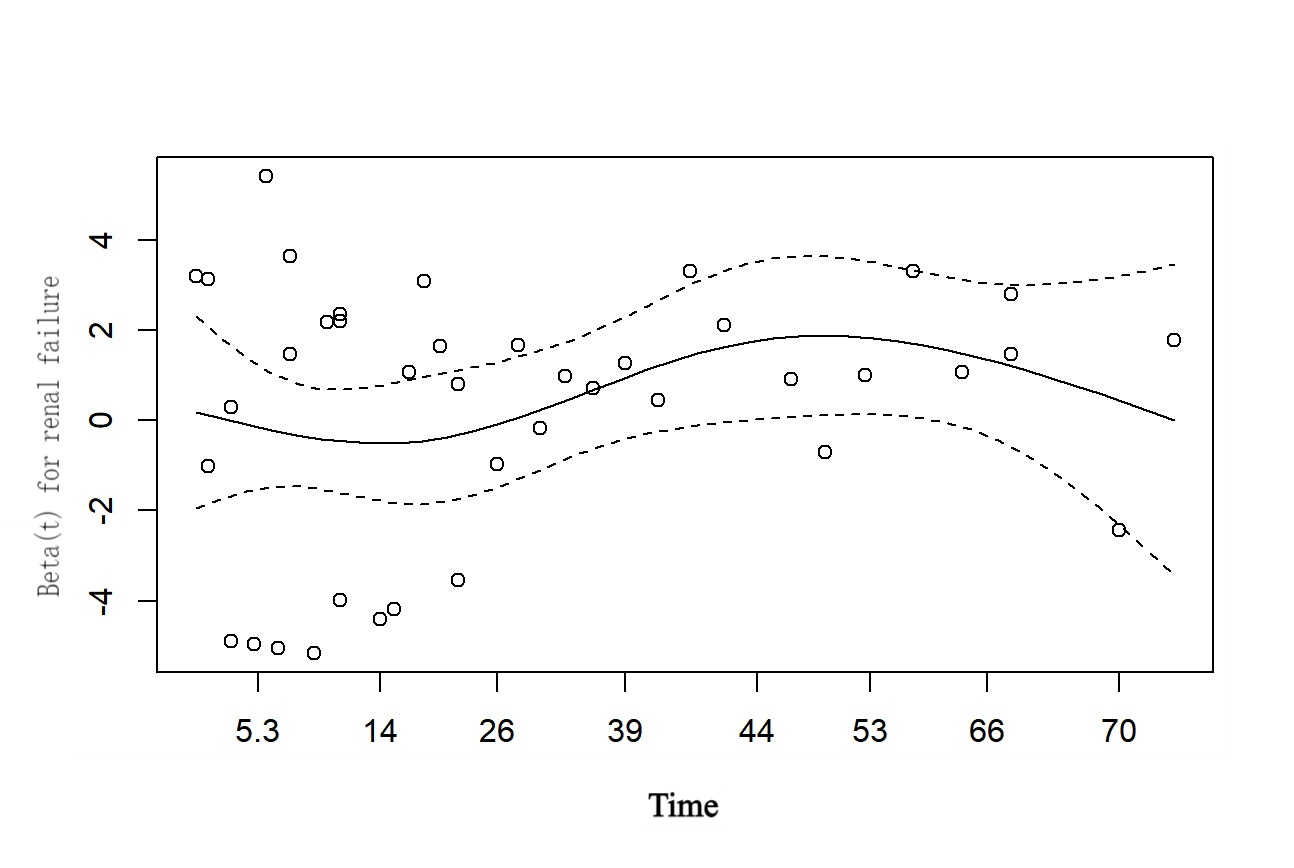

Supplement: Supplementary file 4 [file Image_2.png]
